# An activity-dependent proximity ligation platform for spatially resolved quantification of active enzymes in single cells

**DOI:** 10.1038/s41467-017-01854-0

**Published:** 2017-11-24

**Authors:** Gang Li, Jeffrey E. Montgomery, Mark A. Eckert, Jae Won Chang, Samantha M. Tienda, Ernst Lengyel, Raymond E. Moellering

**Affiliations:** 1 Department of Chemistry, The University of Chicago, Chicago, IL 60637 USA; 2 Institute for Genomics and Systems Biology, The University of Chicago, Chicago, IL 60637 USA; 3 Department of Obstetrics and Gynecology/Section of Gynecologic Oncology, The University of Chicago, Chicago, IL 60637 USA

## Abstract

Integration of chemical probes into proteomic workflows enables the interrogation of protein activity, rather than abundance. Current methods limit the biological contexts that can be addressed due to sample homogenization, signal-averaging, and bias toward abundant proteins. Here we report a platform that integrates family-wide chemical probes with proximity-dependent oligonucleotide amplification and imaging to quantify enzyme activity in native contexts with high spatial resolution. Application of this method, activity-dependent proximity ligation (ADPL), to serine hydrolase and cysteine protease enzymes enables quantification of differential enzyme activity resulting from endogenous changes in localization and expression. In a competitive format, small-molecule target engagement with endogenous proteins in live cells can be quantified. Finally, retention of sample architecture enables interrogation of complex environments such as cellular co-culture and patient samples. ADPL should be amenable to diverse probe and protein families to detect active enzymes at scale and resolution out of reach with current methods.

## Abstract (web summary)

The interrogation of enzyme activity involves the ensemble averaging of many cells, loss of spatial relationships and is often biased to abundant proteins. Here the authors develop activity-dependent proximity ligation to quantify enzyme activity at the cellular and sub-cellular level in relevant biological contexts.

## Introduction

The study of protein function has traditionally been a reductionist endeavor, where proteins are expressed and purified from orthogonal hosts and then studied in isolation. We know, however, that many of the functional properties of a protein are imparted by the complexity of the surrounding environment, including participation in protein–protein complexes^1, 2^, spatial localization to distinct sub-cellular compartments^3, 4^, post-translational chemical modifications^5^, and even mechanical forces within^6, 7^ or between cells^8^. Despite our appreciation for these influences, traditional biophysical and biochemical techniques rarely capture the effects of these events. The field of proteomics aims to provide a comprehensive accounting of the compliment of proteins in a biological sample. In the decade since orbitrap mass spectrometers and analysis algorithms^9^ have become commercially available, the field of proteomics has found mainstream applications in basic chemical, biological, and clinical research^10–12^. Despite the power of these technologies, standard proteomic platforms are typically limited to providing two pieces of information: whether a specific protein is present in a sample, and the relative abundance of a protein within a sample. While this information is important, it does not provide information on the functional state of the detected proteins. Activity-based proteomic technologies, on the other hand, integrate enzyme- or protein-family-specific chemical probes with traditional mass spectrometry or gel-based profiling methods in order to detect and quantify protein activity, rather than abundance^12–14^. These measurements can be made directly with complex samples such as lysate, tissues, and biological fluids to measure changes in protein activity, often for entire families of proteins of a 100 or more^15–17^, that result from endogenous biological signals or the action of exogenous molecules (e.g., therapeutics).

Activity-based profiling approaches and the mass spectrometry platforms upon which they rely have two major limitations. First, gel-based or mass spectrometry-based proteomic experiments impose significant limits on the amount of sample needed, which generally prevents the analysis of limited abundance samples (e.g., patient tissue) and single-cell measurements. Even with ample input proteome, gel-based, and data-dependent LC-MS/MS measurements are heavily biased toward high abundance proteins, often omitting a majority of the proteome in routine analyses^18^. CyTOF^19^ and imaging mass spectrometry^20^ approaches can provide quantitative information on protein abundance with single-cell resolution, however these approaches require expensive mass spectrometry equipment and antibody conjugates, and do not report on protein function. Second, current proteomic methods require homogenization and manipulation of the biological sample, which results in the loss of spatial information about protein activity, both at intra- and intercellular levels. Expression of fluorescent protein-tagged proteins^3^ or the use of proximity ligation assays targeting complexes^21–24^ or modified forms of a protein of interest^25–28^ can provide information on sub-cellular localization, however these approaches often require genetic manipulation, availability of multiple proteoform-specific antibodies, and a priori information correlating functional state with specific proteoforms of a protein. Activity-based probes detect protein activity, but involve loss of spatial information and require significant input proteome. Small-molecule “turn-on” probes^29^ typically lack the ability to provide precise spatial information due to signal diffusion, and sometimes do not reflect activity of a single protein but a protein family. Several recent studies have applied iterative medicinal chemistry and screening to transform non-selective family-wide probes into enzyme-specific reporter probes for lipid hydrolases^30, 31^ and caspase-family cysteine proteases^32^. Through the covalent tagging of active enzymes with a fluorescent reporter, these probes have enabled sub-cellular and intercellular visualization and quantification of active enzymes, in live cells and in vivo. While providing a step forward in chemical proteomics, like “turn-on” probes this approach is hardly general, as each enzyme requires de novo development of tailored chemical probes that exhibit extremely high target selectivity. To address the inherent shortcomings of existing proteomic technologies, we sought to develop a chemical proteomic platform that can, in principle, overcome these limitations. Our goal was to develop a novel platform that could provide three features typically absent in proteomic profiling: (1) Quantification of protein activity and function, rather than abundance; (2) Enable direct visualization of localized enzyme activity at the sub-cellular and intercellular scale; (3) Increased dynamic range through signal amplification to allow measurement of low-abundance proteins and samples. Here we report such a platform, named activity-dependent proximity ligation (ADPL).

## Results

### ADPL quantifies active enzymes with high spatial resolution

**Fig. 1:**
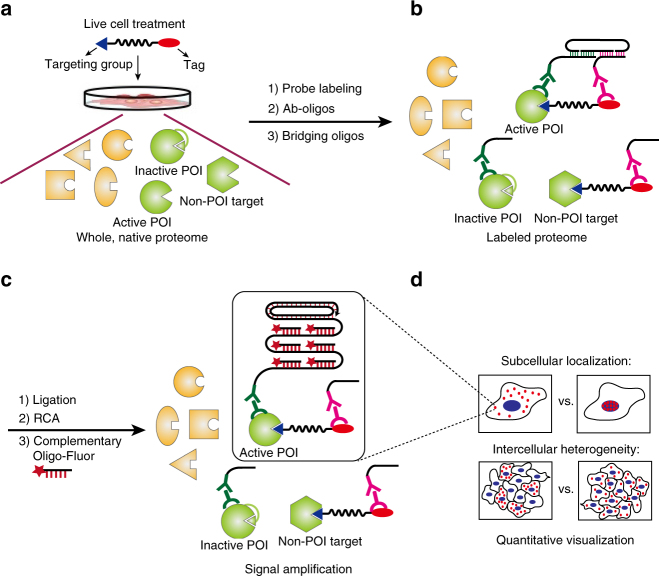
Schematic depicting the activity-dependent proximity ligation (ADPL) workflow. **a** Live cells pulsed with a family-wide chemical probe labels active proteins within their native environment. **b** Detection of probe-labeled protein-of-interest (POI) is accomplished by incubation of fixed cells with primary antibodies directed to the POI, and probe detection handle (biotin). Subsequent incubation with secondary antibody-oligonucleotide conjugates directed against each primary antibody enables hybridization and ligation of two bridging complementary oligonucleotides only when the probe and POI are in high proximity (i.e., on the same protein). **c** Signal amplification and detection is achieved through ligation, rolling circle amplification, and subsequent hybridization of fluorophore-conjugated complementary oligonucleotides. **d** Visualization and quantification of sub-cellular and intercellular enzyme activity is afforded by fluorescence microscopy

ADPL integrates the activity-dependent and family-wide tagging of endogenous, active enzymes afforded by chemical probes, with the specific and robust signal amplification afforded by barcoded oligonucleotide proximity ligation and amplification (Fig. 1)^21^. In contrast to the majority of studies that only use chemical probes in homogenized cell lysate, we sought to tag active enzymes in their native environment, and thus we performed ADPL by pulsing live cells with a family-wide probe (Fig. 1a). Whole fixed cells are then labeled with probe-specific and protein-of-interest (POI)-specific antibodies, and subsequently secondary antibodies conjugated to barcoded, single-stranded oligonucleotide sequences (Fig. 1b). In this way, the chemical probe provides a significant narrowing of the proteome under study, and the POI antibody allows for deconvolution of signal from a family-wide probe, which may have tagged hundreds of proteins, to that from just one protein. Subsequent incubation with sequence-specific bridging oligonucleotides allows for ligation and rolling circle amplification of probe-labeled target proteins (Fig. 1c). Finally, ADPL signal is detected by incubating with a complementary, fluorophore-labeled oligonucleotide and fluorescence microscopy (Fig. 1c, d)^21, 22^. In summary, ADPL seeks to provide a highly specific, selective, amplified fluorescent signal for an active POI within the preserved complex cellular environment.

**Fig. 2:**
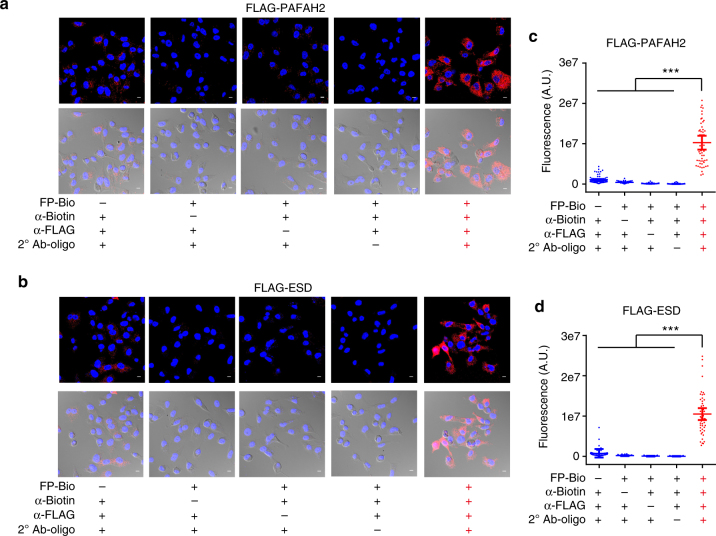
Modular, specific detection of active serine hydrolases by ADPL imaging. **a**, **b** Representative ADPL detection and visualization of active FLAG-PAFAH2 (**a**) and FLAG-ESD (**b**) in PC3 cells in the presence or absence of the indicated ADPL components. Channels shown are DAPI nuclear stain (blue), ADPL signal (red), and overlayed signal on light field images. **c**, **d** Quantified single-cell ADPL fluorescent signal from active FLAG-PAFAH2 (**c**) and FLAG-ESD (**d**) in the presence or absence of indicated ADPL components, demonstrating the probe- and POI-dependent nature of a robust ADPL signal. Quantification of signal in **c**: minus FP-Bio (*n* = 76), minus α-biotin (*n* = 73), minus α-FLAG (*n* = 89), minus 2° antibody-oligo (*n* = 87), positive ADPL (*n* = 53). Quantification in **d**: minus FP-Bio (*n* = 64), minus α-biotin (*n* = 68), minus α-FLAG (*n* = 63), minus 2° antibody-oligo (*n* = 63), positive ADPL (*n* = 50). Unpaired *t-*test results in **c**, **d** are between individual ADPL conditions in the absence of one component and the positive ADPL condition containing all components. ***P < 0.001, Student’s *t* test. Representative images are from triplicate technical replicates of two or more independent biological experiments. Each dot represents a single-cell fluorescence measurement, center line and whiskers denote the mean and 95% C.I. of the population, respectively. Scale bars = 10 μm

**Fig. 3:**
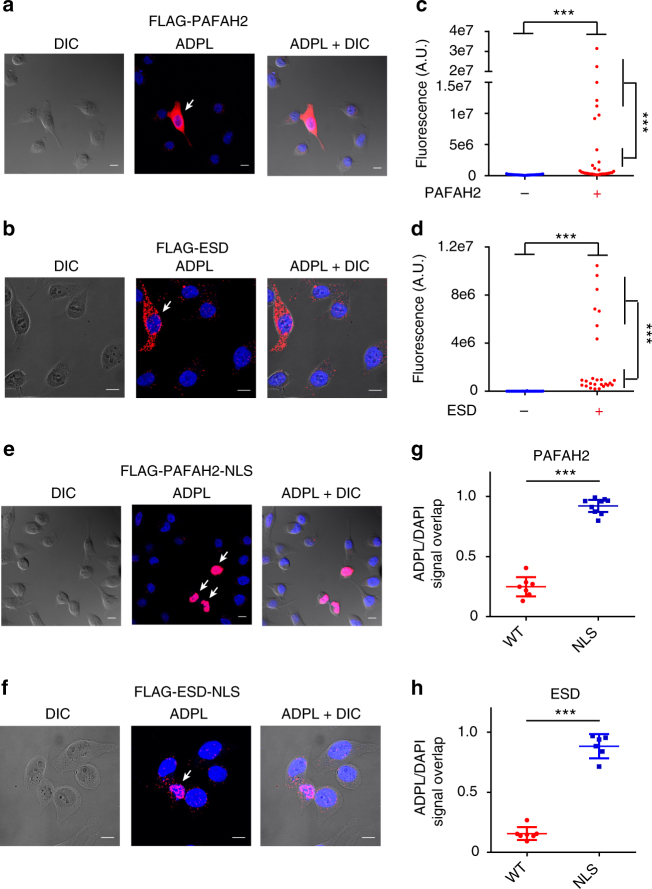
ADPL imaging detects intercellular and intracellular localization of active enzymes. **a**, **b** Representative ADPL images of HeLa cells transiently transfected with FLAG-PAFAH2 (**a**) or FLAG-ESD (**b**); representative outlier cells exhibiting strong ADPL signal and used for quantification of positive cells are denoted by white arrows. **c**, **d** Quantified single-cell ADPL fluorescent signals from representative fields of non-transfected cells and the entire population of cells transfected with FLAG-PAFAH2 (**c**) and FLAG-ESD (**d**). Statistical evaluations shown are comparing mean ADPL signal from positive, transfected cells to the entire field of non-transfected cells (top comparison in both **c**, **d**) and negative cells within the same experiment (right in **c**, **d**). Quantification of signal in **c**: negative transfection (*n* = 31), positive transfection (*n* = 33). Quantification of signal in **d**: negative transfection (*n* = 28), positive transfection (*n* = 26). Denoted “*n*” values indicate total number of cells in each analysis group. **e**, **f** Representative ADPL images of HeLa cells transiently transfected with hydrolases tagged with a nuclear localization sequences: NLS-PAFAH2 (**e**) and NLS-ESD (**f**); representative outlier cells exhibiting strong ADPL signal and used for quantification of positive cells are denoted by white arrows. **g**, **h** Quantification of the ADPL/DAPI fluorescence signal overlay in positive cells, which is a representation of nuclear localization. WT wild-type FLAG-PAFAH2 or FLAG-ESD transfection, as shown in **a**, **b**, respectively. NLS NLS-PAFAH2 or NLS-ESD transfection, as shown in **e**, **f**, respectively. Quantification of signal in **e**: WT (*n* = 7), NLS (*n* = 9). Quantification of signal in **f**: WT (*n* = 7), NLS (*n* = 6). Scale bars = 10 μm in all images. Blue channel: DAPI nuclear; red channel: ADPL signal; gray channel: DIC. ***P < 0.001, Student’s *t* test. Each dot represents a single-cell fluorescence measurement, center line and whiskers denote the mean and 95% C.I. of the population, respectively. Representative images are from triplicate technical replicates of two or more independent biological experiments

To test this approach within a well-characterized enzyme family, we employed a fluorophosphonate-biotin (FP-Bio) chemical probe that covalently modifies active serine hydrolase enzymes, of which there are ~200 in mammalian cells^12, 13^.We first tested whether the ADPL platform could specifically detect the activity of two soluble serine hydrolase enzymes, platelet-activating factor acetylhydrolase 2 (PAFAH2) and esterase D (ESD). PC3 prostate cancer cells stably expressing FLAG-tagged PAFAH2 and ESD were pulsed with FP-Bio and processed for ADPL with an anti-FLAG antibody (Fig. 2a; Supplementary Fig. 1). Cells treated with FP-Bio and fully processed for ADPL exhibited intense fluorescence signal throughout the cytosol, consistent with predicted PAFAH2 and ESD localization (Fig. 2a, b). Omission of any component or step in the ADPL protocol resulted in significant reversion of signal to background. Relative quantification of the ADPL signal for these enzyme targets yielded highly significant signal increases of ~10–250-fold for both PAFAH2 and ESD, relative to background (Fig. 2c, d). To determine whether ADPL could identify and detect distinct cellular phenotypes within a heterogeneous cellular population, PAFAH2 and ESD were expressed in HeLa cells via transient transfection, resulting in mixtures of positive (transfected) and negative expressing (untransfected) cells. ADPL imaging was able to differentiate both PAFAH2-expressing and ESD-expressing cells that, between or within an experiment, exhibited significant increases in signal of ~12–900-fold over negative cells (Fig. 3a–d; Supplementary Fig. 2a–f). Notably, gel-based profiling, which relies on averaging over many thousands of cells, was unable to detect the presence of these outlier cells when a heterogeneous cell population was present (Supplementary Fig. 3a, b), highlighting the ability of ADPL to provide quantitative enzyme activity information at single-cell resolution.

Localization of biomolecules to distinct sub-cellular compartments and complexes can have a significant impact on protein function, however the simultaneous detection of activity and localization is challenging with current approaches. To determine if ADPL could detect the sub-cellular localization of active enzymes, the cytosolic PAFAH2 and ESD enzymes were tagged with a C-terminal nuclear localization sequence (NLS) and transiently expressed in parallel with the wild-type enzymes. A minority of ADPL signal from wild-type PAFAH2 (~25%) and ESD (~16%) overlapped with the DAPI nuclear signal in a central cellular *z*-plane (Fig. 3g, h). In contrast, the vast majority of PAFAH2-NLS (~92%) and ESD-NLS (~88%) ADPL signals were localized to the nuclear compartment, confirming that ADPL provides spatially resolved information on active enzymes (Fig. 3e–h). We speculate that preservation of sub-cellular information is dependent upon the use of cell-permeable activity probes to tag enzymes in their native environments as well as the subsequent coupling of probe and enzyme in signal amplification and detection.

### ADPL quantifies endogenous determinants of enzyme activity

**Fig. 4:**
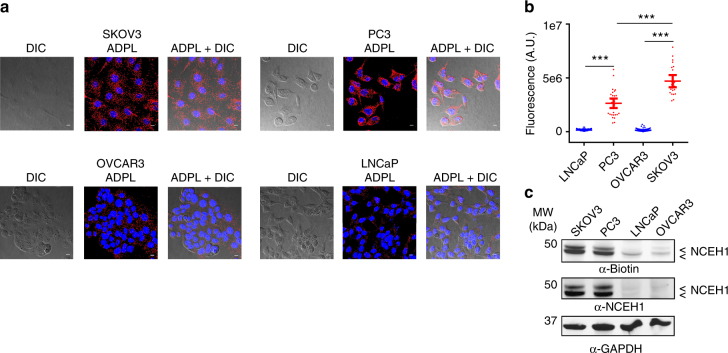
ADPL profiling of differential enzyme activity correlated with distinct phenotypes in native contexts. **a** Representative ADPL images measuring endogenous NCEH1 activity in paired aggressive (SKOV3 and PC3) and non-aggressive (OVCAR3 and LNCaP) cancer cell lines from ovarian and prostate cancers, respectively. **b** Relative quantification of NCEH1 activity in each cell line from **a**. Statistical evaluations shown are comparing mean ADPL signal between non-aggressive and aggressive cells within each tissue of origin. Quantification in **b**: LNCaP (*n* = 47), PC3 (*n* = 43), OVCAR3 (*n* = 60), SKOV3 (*n* = 35). **c** α-Biotin western blot “gel-based” profiling of serine hydrolase activity in the four cell lines is shown. The two bands at ~42 and 45 kDa are glycoforms of NCEH1; the overlapped intermediate band is another enzyme family member. α-NCEH1 immunoblotting indicates protein abundance. α-GAPDH immunoblotting from the same experiment is shown as a loading control. Scale bars = 10 μm in all images. Blue channel: DAPI nuclear; red channel: ADPL signal; gray channel: DIC. ***P < 0.001, Student’s *t* test. Each dot represents a single-cell fluorescence measurement, center line and whiskers denote the mean and 95% C.I. of the population, respectively. Representative images are from quadruplicate technical replicates of three or more independent biological experiments

We next sought to determine whether this proteomic approach could be used to visualize and quantify endogenous active enzymes in cells. Furthermore, we wondered if ADPL could enable interrogation of enzymes that are resistant to the typical biochemical workflow of orthogonal expression, purification, and isolated study with in vitro assays. Neutral cholesterol ester hydrolase 1 (NCEH1, also known as AADACL1 and KIAA1363) is a single-pass transmembrane, differentially glycosylated serine hydrolase implicated in cholesterol ester^33, 34^ and neutral ether lipid^35, 36^ metabolism. The activity of this enzyme has been studied in membrane homogenates from tissues and cells^37^, however it is an example of an enzyme that has not been studied in isolation with typical in vitro biochemical approaches. Similar to the results obtained with FLAG-tagged enzymes, an ADPL workflow coupling the family-wide FP-Bio probe and anti-NCEH1 antibodies detected active NCEH1 in SKOV3 ovarian cancer cells (Supplementary Fig. 4a, c). Previous studies have shown high NCEH1 activity in aggressive tumor cell lines from diverse tissues, whereas less aggressive cell lines display 10–20-fold lower enzyme activity^30, 35–37^. Additionally, high NCEH1 activity has been correlated with tumorigenicity in primary human breast tumors^38^. We thus profiled paired low- and high-aggressiveness cell lines to determine if ADPL could detect and quantify endogenous changes in enzyme activity that correlate with cellular phenotypes. ADPL signal from active NCEH1 enzymes was found to be significantly higher in the more tumorigenic ovarian (SKOV3) and prostate (PC3) cancer cell lines relative to the less aggressive OVCAR3 and LNCaP cells from the same tissues of origin (Fig. 4a, b)^39^. The qualitative differences between these distinct cell lines were apparent in ADPL images; the mean relative differences in NCEH1 activity between the aggressive/non-aggressive pairs from prostate and ovarian cancer cells were 18- and 35-fold, respectively. By comparison, gel-based profiling of NCEH1 signal generated from whole-cell lysate exhibited mean fold-changes of 7- and 18-fold between these same cell line pairs measured by FP-Bio western blot, which was similar to detected changes in total NCEH1 protein abundance by western blot (Fig. 4c). Gel-based profiling also revealed modest differences in NCEH1 activity between the two aggressive cancer cell lines, with SKOV3 cells exhibiting an ~1.6-fold increase relative to PC3 cells. Indeed, this difference was detected by ADPL, with SKOV3 cells showing a statistically significant difference of 1.8-fold increased NCEH1 activity, compared to PC3 cells. Relative to gel-based profiling, the ability to quantify signal at the single-cell level, compared to roughly 10^6^ cells needed for the profile in 4c, enables interrogation of cell population heterogeneity and detection of distinct phenotypes (e.g., Fig. 3a). Additionally, co-migration of other enzyme family members complicates accurate quantification of gel-based signal to a specific enzyme, which is exemplified by a serine hydrolase that co-migrates with the two glycoforms of NCEH1 (Fig. 4c). Together these data establish that the ADPL workflow captures quantitative endogenous variation in enzyme activity in distinct biological states, and comparison with averaged activity-based profiling gels validates that single-cell ADPL data can quantify differences that range from modest (~1.5-fold) to robust (>10-fold).

To test whether ADPL could be applied to other protein families and probe classes, we also utilized a cell-permeable, family-wide probe targeting cathepsins, a subfamily of cysteine proteases^40^. In particular, we assessed the activity of Cathepsin B in U87 glioblastoma cells by ADPL, and found similar results to those observed for serine hydrolases (Supplementary Fig. 5a, b). Antibody-based immunofluorescent staining of cathepsin B (CTSB) protein revealed signal distributed evenly throughout the cytosol (Supplementary Fig. 5c). ADPL signal from CTSB, in contrast, was more restricted to foci that predominantly co-localized with LAMP1 + lysosomes (Supplementary Fig. 5d, e). These results demonstrate the notion that ADPL simultaneously measures protein activity and location, which in the case of CTSB has been shown to occur primarily in the low pH environment of endolysosomal compartments^41^. Immunofluorescence and other proteomic approaches, on the other hand, indiscriminately report on both active and inactive proteins. These results, and the modular nature of this ADPL platform suggest that ADPL should be applicable to diverse protein families and probe classes.

### ADPL quantifies small-molecule target engagement in cells

**Fig. 5:**
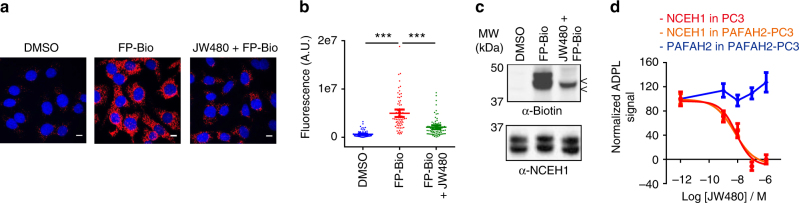
ADPL detects active enzymes and the specific action of small-molecule inhibitors. **a**, **b** Representative ADPL images (**a**) and quantification (**b**) of endogenous NCEH1 activity in SKOV3 cells with no FP-Bio probe treatment (DMSO), with FP-Bio and with FP-Bio after pre-treatment of live cells with the specific NCEH1 inhibitor JW480 (1 μM, 4 h). Quantification in **b**: DMSO (*n* = 73), FP-Bio (*n* = 73), FP-Bio + JW480 (*n* = 79). **c** α-biotin western blot “gel-based” profiling of NCEH1 activity from conditions in **a** show that while NCEH1 is present in all conditions, JW480 specifically inhibits labeling of NCEH1 by FP-Bio. **d** Quantification of NCEH1 activity by ADPL in wild-type or PAFAH2-expressing PC3 cells treated with JW480 prior to ADPL imaging. IC_50_ curves exhibit potent and precise inhibition of NCEH1 by JW480 in wild-type PC3 cells (IC_50_ = 6 nM) and PAFAH2-expressing PC3 cells (IC_50_ = 8 nM). Parallel quantification of PAFAH2 activity in the stable PC3 cell line shows no effect of JW480 over the same dose range. In **b**, each point represents a single-cell fluorescence measurement, center line and whiskers denote the mean and 95% C.I. of the population, respectively. Sigmoidal IC_50_ curves in **d** were generated in Prism 6 software, with center lines and error bars denoting mean and s.e.m. Scale bars = 10 μm in all images. Blue channel: DAPI nuclear; red channel: ADPL signal; gray channel: DIC. ***P < 0.001, Student’s *t* test. Data are from quadruplicate technical replicates in two or more biological experiments

A key advantage of activity-based probes is their dependence upon the catalytic integrity of target proteins. This requisite connection between protein activity and probe signal enables the quantification of endogenous changes in protein activity, e.g., caused by post-translational modification of a given target, as well as the action of exogenous agents, such as small-molecule drugs^12, 42^. To understand whether ADPL is indeed reporting on the activity of target proteins, rather than abundance, we sought to detect and quantify the effects of small-molecule inhibitors with ADPL. First, SKOV3 cells were treated with 1 μM of an NCEH1-selective small-molecule inhibitor, JW480^36^, prior to pulse labeling with FP-Bio and ADPL processing. NCEH1 activity in JW480-treated cells was significantly reduced relative to those treated with vehicle alone, which was apparent by both ADPL imaging and quantification (Fig. 5a, b). Parallel gel-based profiling from homogenized cells likewise revealed significant and selective inhibition of NCEH1 activity with JW480 treatment, despite equivalent NCEH1 protein levels across these conditions (Fig. 5c). Treatment of cells with JW480 also demonstrated dose-dependent inhibition of NCEH1, with an apparent IC_50_ = 6 and 8 nM in PC3 and PAFAH2-expressing PC3 cells, respectively (Fig. 5d). These IC_50_ values were very similar to those previously reported by gel-based profiling under slightly different conditions in PC3 cells^36^. To confirm that the inhibitory action we observed was specific to NCEH1, we monitored the activity of PAFAH2 in parallel; no inhibition of target signal was observed in response to JW480 (Fig. 5d; Supplementary Fig. 6a, b). These data confirm that ADPL can detect graded changes in enzyme activity in response to both endogenous and exogenous activity modulators. Furthermore, this approach offers a general way to detect and quantify target engagement in live cells, particularly for enzyme targets that are resistant to traditional in vitro approaches, such as post-translationally modified, insoluble enzymes like NCEH1.

### ADPL quantifies phenotypic heterogeneity in patient tissues

**Fig. 6:**
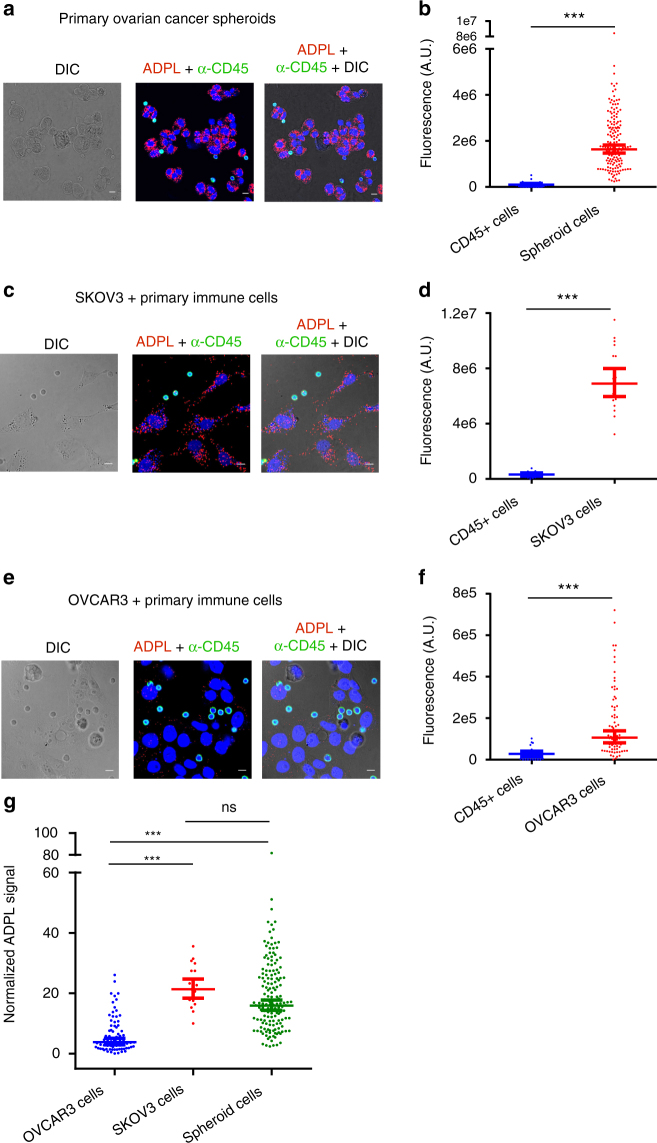
ADPL quantification of endogenous enzyme activity in cellular co-culture and primary patient samples. **a**, **b** Representative ADPL images (**a**) and quantification (**b**) of NCEH1 activity in primary ovarian cancer spheroids. Simultaneous CD45 staining (green) marks immune cells present in heterogeneous spheroids. Quantification in **b**: immune cells (*n* = 25), cancer cells (*n* = 155). **c**, **d** Representative ADPL images (**c**) and quantification (**d**) of NCEH1 activity in cellular co-culture of aggressive SKOV3 ovarian cancer cells and primary immune cells. Quantification in **d**: immune cells (*n* = 16), cancer cells (*n* = 20). **e**, **f** Representative ADPL images (**e**) and quantification (**f**) of NCEH1 activity in cellular co-culture of non-aggressive OVCAR3 ovarian cancer cells and primary immune cells. Quantification in **f**: immune cells (*n* = 23), cancer cells (*n* = 74). **g** Normalized ADPL signal of OVCAR3, SKOV3, spheroid cancers cells relative to co-cultured CD45 + immune cells (data in **b**, **d**, **f**). Scale bar: 10 μm. Blue channel: DAPI; red channel: ADPL; green channel: CD45; gray channel: DIC. Each point represents a single-cell fluorescence measurement, center line and whiskers denote the mean and 95% C.I. of the population; unpaired Student’s *t* test was used for statistical significance. ***P < 0.001; ns not significant, Student’s *t* test. Data are from four or more technical replicates from independent duplicate biological experiments

One of the challenges with both traditional and activity-based proteomic approaches is determining whether the averaged signal observed by gel or LC-MS/MS-based detection is representative of the population being studied^43^. Due to the retention of cellular structure and reporting of activity from single cells, we hypothesized that ADPL could detect and quantify active enzymes in biologically-relevant, heterogeneous environments such as cellular co-culture. Furthermore, we sought to test whether ADPL could be used to probe enzyme activity in complex primary tissue samples, such as individual patient-derived, ovarian cancer spheroids. These organoid tissues are heterogeneous mixtures of cells often detected in ascites^44^, as well as other tumor types^45^. Despite the significance of these organoids in disease, standard mass spectrometry or gel-based methods cannot be used to study protein abundance or activity due to their small size (~100s of cells). Given the established relationship between NCEH1 activity and ovarian cancer cell aggressiveness, we applied ADPL to detect and quantify active NCEH1 in cellular co-culture and patient-derived spheroids. Image-based ADPL quantification of NCEH1 activity in dissociated individual spheroids revealed that they were not homogeneous and instead consisted of both ovarian cancer cells and CD45^+^ immune cells (Fig. 6a). NCEH1-dependent ADPL signal was almost entirely localized to the ovarian cancer cells relative to CD45^+^ cells, quantified as an ~20-fold and ~7-fold increase in raw and area-normalized NCEH1 ADPL activity, respectively (Fig. 6a, b; Supplementary Fig. 7). To determine if these patient-derived cells were more similar to aggressive or non-aggressive ovarian cancer cell lines, we quantified NCEH1 activity in a co-culture system of CD45^+^ lymphocyte monocyte immune cells and aggressive SKOV3 or non-aggressive OVCAR3 cancer cells. Consistent with ADPL experiments on these cell lines alone (Fig. 4a), NCEH1 activity was almost exclusively present in the SKOV3 cancer cells, quantified as an ~22-fold increase in NCEH1 activity relative to immune cells (Fig. 6c, d), whereas much less signal was present in both OVCAR3 and its co-cultured immune cells, likewise quantified as a ~6-fold increase relative to immune cells (Fig. 6e, f). Using the immune cells as a standard we generated a ratiometric “aggressiveness index,” enabling direct comparison of phenotypes in these distinct cellular contexts. These data show significantly increased NCEH1 activity in aggressive SKOV3 and primary ovarian cancer spheroid cells, relative to non-aggressive OVCAR3 cells. These data suggest that the primary ovarian cancer spheroid cells are similar to aggressive, metastatic cells (Fig. 6g), which fits with their annotation as an early stage in ovarian cancer metastasis.

## Discussion

Our goal here was to develop a general chemical proteomic platform to address several shortcomings that plague current proteomic profiling approaches. Chief among these were the inability to probe a wide dynamic range of sample abundance, provide information on the functional state of proteins, and the capacity to quantify this information with spatial resolution at the inter- and intracellular scale. Compared to existing activity-based proteomic approaches with gel or LC-MS/MS as a readout, the incorporation of a specific and robust amplification scheme applied in native cell environments allows for significant expansion of the questions that can be addressed in biological systems. First, ADPL permits quantification of enzyme activity across a high dynamic range with respect to sample input as well as relative abundance within the proteome of a given cell. Here we demonstrated that this aspect allows for single-cell resolution, as well as interrogation of low abundance or low-activity protein targets, both of which represent important contexts in biology. Single-cell resolution and low-sample requirements enabled the detection and quantification of enzyme activity in heterogeneous cellular populations, including cellular co-culture and primary ovarian cancer spheroids. The fact that ADPL does not require any genetic manipulation is also important to allow for direct compatibility with other types of primary tissues and fluids. The use of proximity-dependent, barcoded oligonucleotides for signal amplification suggests that other readouts besides fluorescence imaging, such as quantitative PCR and sequencing, should be possible. Additionally, implementation of barcoded oligo-fluorophores or primers should enable multiplexed readout of active enzymes within and between families, as well as integration with methods to simultaneously capture information on transcript and protein abundance. The use of cell-permeable family-wide chemical probes permits tagging of active proteins in their native cellular context, which we posit provides a better representation of their functional properties^46^. Here we established the applicability of ADPL to serine hydrolase enzymes under external (ESD and PAFAH2) or endogenous regulation (NCEH1 and fatty acid amide hydrolase, FAAH, Supplementary Fig. 4b, d). Additionally, we showed that ADPL can be extended to other classes of chemical probes and corresponding enzyme families, such as the cysteine protease CTPB. Within this study, we chose biotin for a recognition moiety for several reasons, including the availability of binding reagents (e.g., antibodies and streptavidin), the high affinity and specificity of these binding interactions, and the ability to generate cell-permeable chemical probes. Endogenous biotinylated proteins may play a role in background signal in this and other ADPL formats, and therefore future exploration of other recognition moieties, both chemical^47^ or orthogonal receptor-based, is warranted. Since this technique does not require sample homogenization, it allows for retention of quantitative, activity-dependent information at the inter- and intracellular scale. We anticipate that these aspects of ADPL imaging will be important to study the relationship between protein abundance, localization, and activity in a variety of biological contexts (e.g., cancer, inflammation, immune function, development).

Another powerful aspect of this platform is its ability to directly probe enzyme activity in living cells, obviating the need to develop specific activity assays and the process of overexpression and purification for a target protein of interest. Indeed, for many proteins, such as the glycosylated, integral membrane hydrolase NCEH1 studied here, this workflow may not be possible at all. ADPL was used to detect endogenous differences in enzyme activity among distinct cellular phenotypes, as well as to interrogate the action of small-molecule inhibitors on enzyme function directly in live cells. This process only required the knowledge that the enzyme is targeted by the family-wide probe, and the availability of a single antibody for the protein of interest. In the case of PAFAH2, genetic incorporation of a modular affinity tag allowed for activity measurements in live cells, indicating that this approach may allow for the development of targeted assays for enzymes that are known to belong to a specific enzyme family, but do not have known endogenous substrates or are problematic for in vitro biochemistry. Additionally, this approach could be used to verify target engagement in cells without relying on downstream peripheral biomarkers, an important capability in both basic and translational research^12, 14, 42, 48^. Importantly, while we expect that ADPL will expand the biological questions that can be asked with functional proteomics, it is not a replacement for traditional ABPP approaches. The use of activity-based probes coupled to LC-MS/MS shotgun profiling is powerful because it is target agnostic, enabling discovery of active proteins without a priori knowledge of targets^12, 13, 49^. For many applications, ADPL should offer an alternative to the laborious process of developing target-selective chemical probes to overcome issues with detection on gel- and LC-MS/MS platforms, to capture single-cell or spatial information, and to interrogate targets in live cells^43^. Indeed, here we utilized a single, family-wide probe to provide spatially resolved, target-specific information for several diverse serine hydrolases without any optimization. This modularity should extend to other mechanism-driven or affinity-based probes, greatly expanding the information that can be captured on these proteins with spatial resolution, high dynamic range, and in native environments. We therefore expect that this approach, as well as future-related technologies, will enable the interrogation of important basic and translational questions in biology and medicine.

## Methods

### Cell culture

HeLa, PC3, LNCaP, SKOV3, MCF7, and U87 and OVCAR3 cell lines were obtained from ATCC and were not STR profiled. Cell lines have been tested for mycoplasma contamination. HeLa, LNCaP, PC3, SKOV3, MCF7, and U87 cells were cultured in RPMI 1640 (Hyclone, #SH30027.01) supplemented with 10% fetal bovine serum (FBS, Atlanta Biologicals, #912850) and 1% Penicillin/Streptomycin (Hyclone, #SV30010). OVCAR3 and PC3 cells were cultured in DMEM (Hyclone, #SH30243.01) supplemented with 10% FBS, 1% Penicillin/Streptomycin, 1% MEM nonessential amino acids (Corning, #25-025-CI), and 1% MEM vitamins (Corning, #25-020-CI). All cell lines were grown at 37 °C in a 5% CO_2_ humidified incubator.

### Family-wide probes

Fluorophosphonate-biotin probe synthesis: To synthesize the probe FP-biotin (FP-Bio), precursors 1 and 2 (Supplementary Fig. 8) were synthesized according to the previous published procedures^15, 50^. Precursor 1 (41 mg, 0.1 mmol, 1.0 equivalent) and DIPEA (70 μL, 0.4 mmol, 4.0 equivalent) were dissolved in DMF (0.4 mL, 0.25 M) at room temperature. Precursor 2 (40 mg, 0.12 mmol, 1.2 equivalent) was then added and the mixture was stirred overnight, and concentrated under reduced pressure. The crude material was purified by column chromatography (2–12% MeOH/DCM gradient) to give FP-Bio as a white solid. (18 mg, 28.9%). ^1^H NMR (500 MHz, CDCl_3_) *δ* 6.16 (s, 2H), 5.35 (s, 1H), 4.90 (s, 1H), 4.52 (dd, *J* = 7.4, 5.1 Hz, 1H), 4.33 (dd, *J* = 7.4, 4.8 Hz, 1H), 4.26 (m, 2H), 4.02 (t, *J* = 6.5 Hz, 2H), 3.23 (dd, *J* = 12.7, 6.6 Hz, 2H), 3.16 (m, 3H), 2.92 (dd, *J* = 12.8, 4.9 Hz, 1H), 2.77–2.71 (m, 1H), 2.21 (t, *J* = 7.2 Hz, 2H), 1.95–1.83 (m, 2H), 1.80–1.25 (m, 31H). ^13^C NMR (126 MHz, CDCl_3_) *δ* 173.30 (s), 164.19 (s), 156.19 (s), 64.97 (s), 63.17 (d, *J* = 7.3 Hz), 61.91 (s), 60.27 (s), 55.64 (s), 40.66 (s), 39.34 (s), 35.95 (s), 30.43 (s), 30.30 (s), 29.74 (s), 29.50 (s), 29.31 (s), 29.27 (s), 29.15 (d, *J* = 4.9 Hz), 29.03 (s), 28.13 (d, *J* = 7.0 Hz), 25.95 (s), 25.70 (s), 25.07 (s), 24.84 (s), 23.91 (s), 23.69 (s), 21.98 (d, *J* = 5.5 Hz), 16.47 (d, *J* = 5.6 Hz). HRMS (*m/z*): [M]^+^ calcd. for C_28_H_52_FN_4_O_6_PS, 622.3329; found 622.3342.

The cathepsin family-wide probe was obtained from ActivX Biosciences (AX13146).

### Immunoblotting

Cells were harvested by scraping in PBS, pelleted by centrifugation at 1000 rpm, washed twice with PBS and lysed in PBS (pH 7.4) containing complete protease inhibitor cocktail (Sigma, #92714-1BTL) by sonication at 4 °C. Protein concentration was determined by BCA assay (Pierce, #23225); the cell lysate was diluted into 4× Laemmli buffer (4×: 200 mM Tris pH 6.8, 400 mM DTT, 8% SDS, 0.4% bromophenol, 40% glycerol), followed by heating to 95 °C for 5 min, cooling to room temperature, and gel electrophoresis on NuPAGE Novex 4–12% Bis-Tris Protein Gels (Invitrogen, NP0322BOX). PAGE gels were transferred onto nitrocellulose membranes, blocked in 2% BSA in TBS containing 0.1% tween-20 (TBST) and probed with primary and secondary antibodies. Primary antibodies used in this study include: anti-FLAG-M2 (1:2000, F1804, Sigma Aldrich), anti-NCEH1 (in-house mouse polyclonal 1:2000 from 1 mg/mL stock), anti-GAPDH (1:2000, Cell Signaling Technology, #2118 S). Blots were imaged using fluorescence-labeled secondary antibodies, IRDyeR-800CW anti-rabbit (LI-COR, #926-32213) or IRDyeM-680RD anti-mouse (LI-COR, #926-68072), on the OdysseyCLxImager (LI-COR). Quantification of band intensities has been performed using ImageJ software (NIH).

### ESD-NLS and PAFAH2-NLS plasmid construction

Full-length, human ESD (NM_001984), and PAFAH2 (NM_000437) in pCMV6 entry vectors with C-terminal Myc-DDK tag were purchased from Origene. The ESD-NLS and PAFAH2-NLS were generated according to a previously published procedure^51^. Briefly, TagMaster mutagenesis kit (GM Biosciences, #GM7002) was employed to introduce a C-terminal SV40 NLS (PKKKRKV) between the existing DDK tag and stop codon in the pCMV6 entry vector. Mutagenesis was performed according to manufacturer’s protocol with the following primers:

Forward: 5′-AAGGATGACGACGATAAGCCGAAGAAGAAGCGCAAGGTGGTTTAAACGGCCGGCC-3′;

Reverse:

5′-GGCCGGCCGTT TAAACCACCTTGCGCTTCT TCTTCGGCTTATCGT CGTCATCCTT-3′. The resulting ESD-NLS and PAFAH2-NLS constructs were used for transient transfection experiments in HeLa cells.

### Transient transfection

About 4 × 10^5^ HeLa cells were seeded in six-well plate. About 16 h later, transfection was performed when the cells reached to 60–80% confluency. In terms of the transfection, 200 ng plasmid was added to 60 μL serum free RPMI 1640 medium and 2.5 μL lipofectamine 2000 (Thermo Fisher, #11668027) was added to 60 μL serum-free RPMI 1640 medium. The two solutions were incubated at room temperature for 10 min. Then, the plasmid was added to the lipofectamine solution and the mixture was incubated at room temperature for 15 min, followed by addition of 480 μL serum free medium. The cells were washed with PBS and then transfection mixture was added. After 4 h transfection, the medium was changed to normal medium. About 24 h later, the cells were trypsinized and seeded into 12-well chamber slide (Ibidi, #81201). ADPL was run according to the protocol below.

### Immunofluorescence sample preparation

Cells were seeded in 12-well chamber slide. About 12–24 h later, when reaching 80–90% confluency, the cells were fixed with 4% paraformaldehyde in PBS at room temperature for 15 min, washed twice with PBS for 5 min each with orbital shaking. Cells were permeabilized in 0.5% Triton X-100 (Fisher) in PBS at room temperature for 15 min, washed twice with 0.05% Tween-20 (Fisher) in PBS for 5 min each at room temperature with orbital shaking. The chamber was removed and the well boundary was delineated with the hydrophobic barrier pen (Vector laboratories, #H-4000). One-drop Duolink blocking buffer (Sigma, #DUO92004) was added and the slide was incubated at 37 °C for 30 min in a humidified chamber. Anti-FLAG antibody (mouse, 1:100, final concentration 10 μg/mL) was added to the wells and incubated overnight at 4 °C. The slide was washed in TBST buffer three times for 5 min each. Oregon Green 488 goat anti-mouse (Invitrogen, #011033, final concentration 10 μg/mL) was added to the wells and incubated for 1 h at 37 °C, followed by washing in TBST buffer three times. The slide was mounted in mounting buffer (ProLong Gold, Thermo Fisher Scientific, #P10144) and used for confocal fluorescence microscopy.

### Lentiviral expression vector cloning

To generate lentiviral vectors for constitutive expression, PAFAH2 and ESD were cloned into the pLenti6 backbone. pLenti-6-TP53-R273H (Addgene, #22934) was digested with BamH1-HF (New England BioLabs, #R3136S) and Age1-HF (New England BioLabs, #R3552S) and extracted with phenol–chloroform. Blunt ends were created using DNA polymerase I, large (Klenow) fragment (New England BioLabs, #M0210S), followed by phenol–chloroform extraction. Antarctic phosphatase (New England BioLabs, #M0289) was used to dephosphorylate the 5′ and 3′ ends. Following electrophoresis (0.8% agarose), linearized backbone was excised and frozen. DNA was eluted through a polyethylene filter and phenol–chloroform extracted.

PAFAH2 (Origene, #RC200355) and ESD (Origene, #RC200533) constructs were digested using EcoR1-HF (New England BioLabs, #R3101S) and Fse1 (New England BioLabs, #R0588S), followed by heat inactivation. Blunt ends were created using DNA Polymerase I, large (Klenow) fragment (New England BioLabs, #M0210S). Following electrophoresis (0.8% agarose),the linearized insert was excised and frozen. DNA was eluted through a polyethylene filter and phenol–chloroform extracted.

Backbone and insert were ligated using T4 DNA ligase (New England BioLabs, #M0202). NEB 5-alpha competent *Escherichia coli* (high efficiency) cells (New England BioLabs, #C2987I) were transformed with the ligated plasmid. Transformed bacteria were plated on LB + Amp (100 µg/mL) agar plates and incubated at 37 °C overnight. Plasmid sequences were verified with Sanger sequencing at the University of Chicago Comprehensive Cancer Center DNA Sequencing Facility using CMV-f and pBABE-r primers. Forward sequencing primer: CMV-f 5′-CGCAAATGGGCGGTAGGCGTG-3.′ Reverse sequencing primer: pBABE-r 5′-ACCCTAACTGACACACATTCC-3′.

### Stable cell line generation

293T cells were seeded in 6 cm dishes (BD Biosciences, #353004) at 1.0 × 10^6^ cells per dish and transfected after 24 h with transfer plasmid (1 µg PAFAH2 or ESD in pLenti6) and packaging vectors (0.1 µg pCMV-VSV-G, Addgene #8454; 0.9 µg pCMV-dR8.2, Addgene #12263) using Lipofectamine 2000 (Invitrogen #11668027). Following overnight transfection, media was exchanged and allowed to incubate for an additional 24 h. Viral collection was performed at 24, 48, and 72 h. Viral media was filtered with a Millex-AA 0.8 µm filter (Fisher Scientific #SLAAV255F) and Polybrene (Sigma #H9268) was added to a concentration of 8 µg/mL before infection of target cell lines. SKOV3 and PC3 cell lines were infected with 48-h viral harvest. After 24 h, cells were allowed to recover by exchanging the media. Cells were selected with Blasticidin (Fisher Scientific #20-335-025MG) at 5 µg/mL for the first three passages as a lower stringency selection. Then, 20 µg/mL was employed as a higher stringency for the following three passages.

### Gel-based activity profiling

Cells were grown in six-well plates or 6-cm dishes until reaching 80–90% confluence. FP-biotin (FP-Bio, 10 mM stock in DMSO) was diluted to 2 μM in DMEM, and added to cells at 37 °C for 40 min. Cells were then washed in PBS, scraped and lysed by sonication using PBS buffer supplemented with protease inhibitor cocktail (Sigma, #92714-1BTL). Protein concentration was determined by BCA assay (Pierce, #23225), lysate was diluted in Laemmli buffer (4×: 200 mM Tris pH 6.8, 400 mM DTT, 8% SDS, 0.4% bromophenol, 40% glycerol), heated to 95 °C for 5 min, and resolved on a 4–12% PAGE gel (Thermo Fisher, NP0322BOX). PAGE gels were processed for western blot as indicated above with IR800-conjugated streptavidin (LI-COR, #926-32230) overnight at 4 °C. Images were captured by Odyssey CLx imaging system (LI-COR). Quantification of band intensities was performed using ImageJ software (NIH).

### Activity-dependent proximity ligation

Cells were seeded in the 12-well chamber slide, typically at 10,000–30,000 cells per well (Ibidi, # 81201). To get an even distribution of the cells, the chamber slide was pre-wetted with cell culture medium, drained off, and the chamber was left at room temperature for 5–10 min after seeding. Cells at 80–90% confluency were pulse treated with either FP-Bio (2 µM) in DMEM and incubated at 37 °C for 40 min or Capthepsin probe (5 µM) in complete medium and incubated at 37 °C for 3 h. Cells were washed with PBS, fixed with 4% paraformaldehyde in PBS at room temperature for 15 min, washed twice with PBS for 5 min each at room temperature with orbital shaking and then permeabilized in 0.5% Triton X-100 in PBS at room temperature for 15 min. Finally, cells were washed twice with 0.05% Tween-20 in PBS for 5 min each at room temperature with orbital shaking.

Prior to antibody incubation, the chamber was removed and the well boundaries delineated with the hydrophobic barrier pen (Vector laboratories, #H-4000). One-drop Duolink blocking buffer (Sigma, #DUO92004) was added and the slide was incubated at 37 °C for 30 min in a humidified chamber. The blocking solution was removed by tapping, followed by addition of 10 µg/mL of the anti-biotin (rabbit, Abcam, #G196266) and primary antibody for the protein of interest: anti-FLAG (mouse, 4 µg/mL of Sigma, #F1804-5mg); anti-NCEH1 (mouse, 4 µg/mL of in-house polyclonal), anti-FAAH (mouse, 4 µg/mL of Abcam, #ab54615) for serine hydrolase members. For the cathepsin B, 20 µg/mL of the anti-biotin (rabbit, Abcam, #G196266) and 10 µg/mL of the anti-cathepsin B (mouse from Abcam, #ab58802) were added following the blocking step. Generally, a 20 µL solution of the two primary antibodies per well was incubated at 4 °C overnight with orbital shaking. Primary solution was removed by tapping; the slide was washed in wash buffer A (150 mM NaCl, 10 mM Tris, 0.05% Tween-20, pH 7.3) three times for 5 min with gentle orbital shaking. Oligo-linked secondary antibodies were then diluted five-fold in antibody diluent buffer (Duolink anti-mouse minus and anti-rabbit plus from Sigma; #DUO92004 and #DUO92002), added to the slide and incubated at 37 °C for 1 h with orbital shaking.

The secondary antibody-probe solution was removed by tapping the slide, followed by washing in buffer A three times with gentle orbital shaking. Ligation mixture (Sigma, Duolink In Situ Detection Reagents Orange kit, #DUO92007) was diluted five-fold in water prior to addition of ligase at a 40-fold dilution. The ligation mixture was incubated at 37 °C for 30 min with orbital shaking, removed, and the slide was washed twice. Finally, amplification solution was diluted five-fold in water prior to addition of polymerase at 80-fold dilution. This amplification solution was added to each well, incubated at 37 °C for 90 min in the dark, and removed by washing with buffer B (0.1 M NaCl, 0.2 M Tris, pH 7.3) twice for 10 min each, followed by washing with 100-fold dilution of wash buffer B for 1 min. Slides were dried at room temperature in the dark, mounted with 50 µL anti-fade mounting solution (Life technology, #P36961), covered with the cover glass (Fisher, #12–545 M), and sealed with nail polish.

For the characterization of the location of cathepsin B in U87 cells, the above ADPL procedure was followed until the amplification step. After wash with buffer B twice for 10 min each, the slides were incubated with either Oregon Green 488 goat anti-mouse (Invitrogen, #011033, final concentration 10 μg/mL) at 4 °C overnight for immunofluorescence, or Alexa Fluor 647 anti-human LAMP1 Antibody (BD, #522622, five-fold dilution) at 4 °C overnight for co-localization study. Then the slide was washed with buffer A twice for 5 min each, followed by washing with 100-fold dilution of PBS for 1 min. Slides were sealed following the procedure above.

### Confocal fluorescence microscopy imaging

Leica SP8 Laser Scanning Confocal was used to image a single focal plane to accurately detect the ADPL signal location using HyD detectors. An Olympus “live cell” DSU Spinning Disk Confocal microscope was employed to get the integrated *z*-stack images to accurately quantify the ADPL signal intensity in Fig. 5a. Identical microscope acquisition parameters were set and used within experiments. Post-acquisition processing was performed using ImageJ software (NIH).

### ADPL image processing and quantification

ImageJ was used to process all images. Lossless TIFF files were employed to quantify fluorescence intensity. To simplify the image processing workflow, a Macro script to automatically process all images was created. The workflow was as follows: open all channels for each field of view; designate a color for each channel; adjust brightness/contrast for all channels (applying the same levels for all conditions within and between experiments to allow for direct comparison); merge the channels together; adjust the image unit from pixel to micrometer; add scale bars; export the processed TIFF files for quantification.

For quantitative analysis, single-cell boundaries were identified manually using the DIC image. Then the “ROI Manager” tool in ImageJ was utilized to add all the cell outlines as a collection and overlay with the ADPL channel to measure per-cell fluorescence intensity. Typical quantitative comparisons were made using data from three or more independent fields of view per independent biological replicate condition.

### Inhibitor profiling by ADPL

Confluent (80–90%) PC3 cells or PAFAH2-expressing PC3 cells were treated with indicated final concentrations of JW480 (0 nM, 1 nM, 10 nM, 100 nM, 1 µM) in complete cell culture medium for 4 h at 37 °C, prior to FP-Bio probe (2 µM) labeling at 37 °C for 40 min in serum-free medium. ADPL workflow was followed as indicated above. Normalized ADPL signal based on no JW480 treatment was created. IC_50_ curves for NCEH1 were generated in Graphpad Prism 6 using the non-linear regression and dose–response inhibition and the connecting curve for PAFAH2 was generated simultaneously.

### ADPL imaging of ovarian cancer spheroids and co-culture

Ovarian cancer spheroid cells were isolated^52^ from the ascites of patients undergoing primary tumor debulking at the University of Chicago Comprehensive Cancer Center with informed consent and with University of Chicago Institutional Review Board approval. Ascites fluid was centrifuged at 3000 rcf for 5 min and resuspended in PBS. Spheroids were collected by passing spheroid suspension through 40 µm nylon mesh (Fisher Scientific, 22363547) and washed thoroughly with PBS. Enriched spheroids were collected from the top of the filter in DMEM growth media and transferred to ultra-low attachment plates (Corning 07-200-601) until seeding. Before seeding the cells, the chamber slide was pre-coated with fibronectin (1:50 from 1 mg/mL stock) for 30 min at room temperature. As a heterogeneous mixture of cells, spheroids cells were seeded directly without cell counting. Two-fold and four-fold dilutions were tried simultaneously for proper confluency at the point of probe treatment. Then, typical ADPL procedure was performed through the rolling circle amplification and detection step. After the slide was washed in wash buffer B, the slide was washed in TBST three times for 5 min and blocked again for 30 min at 37 °C. The anti CD45-FITC (BD Biosciences, #555482; 1:50 dilution) was added to the wells and incubated overnight at 4 °C. The slide was then washed in TBST three times for 5 min and dried at room temperature in the dark, mounted with 50 µL anti-fade mounting solution, covered with cover glass, and sealed with nail polish.

### Co-culture of SKOV3, OVCAR3, and immune cells

Peripheral blood was collected from patients with informed consent (IRB 13372) into purple-cap vacutainers (K_2_EDTA; BD Biosciences, 367861) and peripheral blood mononuclear cells isolated with Ficoll-Paque PLUS (GE Healthcare, 17-1440-02) using manufacturer’s recommended protocol. Before seeding the cells, the chamber slide was pre-coated with fibronectin (1:50 from 1 mg/mL stock) for 30 min at room temperature. Then 8000 SKOV3 and 40,000 CD45^+^ immune cells, or 30,000 OVCAR3 and 10,000 immune cells were seeded in the chamber slide. The same procedure as spheroid cells was adopted in the following steps.

### Statistics statements

All experiments consisted of at least three independent replicates, with biological or technical replicates indicated. All center values given refer the mean and error bars shown represent the standard error of the mean, unless otherwise stated. Sigmoidal binding curves were applied using Prism software and affinities or IC_50_ values reported represent the mean and the 95% confidence interval. Asterisks in figure legends refer to *P*-value thresholds of <0.05 (*), <0.01 (**), or <0.005 (***) from two-sided Student’s *t* tests. No statistical methods or power calculations were used to determine sample size; however these were kept constant between groups whenever possible.

### Data availability

Primary data and analysis algorithms are available from the authors.

## Electronic supplementary material

Supplementary Information

## References

[1] Agapakis CM, Boyle PM, Silver PA (2012). Natural strategies for the spatial optimization of metabolism in synthetic biology. Nat. Chem. Biol..

[2] Pawson T, Nash P (2000). Protein-protein interactions define specificity in signal transduction. Genes Dev..

[3] Kumar A (2002). Subcellular localization of the yeast proteome. Genes Dev..

[4] Yu CS, Chen YC, Lu CH, Hwang JK (2006). Prediction of protein subcellular localization. Proteins..

[5] Walsh C (2006). Posttranslational Modification of Proteins: Expanding Nature’s Inventory.

[6] Fletcher DA, Mullins RD (2010). Cell mechanics and the cytoskeleton. Nature.

[7] Yu H, Mouw JK, Weaver VM (2011). Forcing form and function: biomechanical regulation of tumor evolution. Trends Cell Biol..

[8] Meloty-Kapella L, Shergill B, Kuon J, Botvinick E, Weinmaster G (2012). Notch ligand endocytosis generates mechanical pulling force dependent on dynamin, epsins, and actin. Dev. Cell.

[9] Eng JK, McCormack AL, Yates JR (1994). An approach to correlate tandem mass spectral data of peptides with amino acid sequences in a protein database. J. Am. Soc. Mass Spectrom..

[10] Walther TC, Mann M (2010). Mass spectrometry-based proteomics in cell biology. J. Cell Biol..

[11] Cox J, Mann M (2011). Quantitative, high-resolution proteomics for data-driven systems biology. Annu. Rev. Biochem..

[12] Moellering RE, Cravatt BF (2012). How chemoproteomics can enable drug discovery and development. Chem. Biol..

[13] Cravatt BF, Wright AT, Kozarich JW (2008). Activity-based protein profiling: from enzyme chemistry to proteomic chemistry. Annu. Rev. Biochem..

[14] Grammel M, Hang HC (2013). Chemical reporters for biological discovery. Nat. Chem. Biol..

[15] Liu Y, Patricelli MP, Cravatt BF (1999). Activity-based protein profiling: the serine hydrolases. Proc. Natl Acad. Sci. USA.

[16] Greenbaum D (2002). Chemical approaches for functionally probing the proteome. Mol. Cell. Proteomics.

[17] Adam GC, Sorensen EJ, Cravatt BF (2002). Proteomic profiling of mechanistically distinct enzyme classes using a common chemotype. Nat. Biotechnol..

[18] Picotti P, Aebersold R, Domon B (2007). The implications of proteolytic background for shotgun proteomics. Mol. Cell. Proteomics.

[19] Bjornson ZB, Nolan GP, Fantl WJ (2013). Single-cell mass cytometry for analysis of immune system functional states. Curr. Opin. Immunol..

[20] Comi TJ, Do TD, Rubakhin SS, Sweedler JV (2017). Categorizing cells on the basis of their chemical profiles: progress in single-cell mass spectrometry. J. Am. Chem. Soc..

[21] Fredriksson S (2002). Protein detection using proximity-dependent DNA ligation assays. Nat. Biotechnol..

[22] Soderberg O (2006). Direct observation of individual endogenous protein complexes in situ by proximity ligation. Nat. Methods.

[23] Gajadhar A, Guha A (2010). A proximity ligation assay using transiently transfected, epitope-tagged proteins: application for in situ detection of dimerized receptor tyrosine kinases. Biotechniques.

[24] Gu GJ (2013). Protein tag-mediated conjugation of oligonucleotides to recombinant affinity binders for proximity ligation. N. Biotechnol..

[25] Robinson PV, Tsai CT, de Groot AE, McKechnie JL, Bertozzi CR (2016). Glyco-seek: ultrasensitive detection of protein-specific glycosylation by proximity ligation polymerase chain reaction. J. Am. Chem. Soc..

[26] Gao X, Hannoush RN (2014). Single-cell in situ imaging of palmitoylation in fatty-acylated proteins. Nat. Protoc..

[27] Elfineh L (2014). Tyrosine phosphorylation profiling via in situ proximity ligation assay. BMC Cancer.

[28] Robinson PV, de Almeida-Escobedo G, de Groot AE, McKechnie JL, Bertozzi CR (2015). Live-cell labeling of specific protein glycoforms by proximity-enhanced bioorthogonal ligation. J. Am. Chem. Soc..

[29] Lemke EA, Schultz C (2011). Principles for designing fluorescent sensors and reporters. Nat. Chem. Biol..

[30] Chang JW, Moellering RE, Cravatt BF (2012). An activity-based imaging probe for the integral membrane hydrolase KIAA1363. Angew. Chem. Int. Ed. Engl..

[31] Chang JW, Cognetta AB, Niphakis MJ, Cravatt BF (2013). Proteome-wide reactivity profiling identifies diverse carbamate chemotypes tuned for serine hydrolase inhibition. ACS Chem. Biol..

[32] Puri AW, Broz P, Shen A, Monack DM, Bogyo M (2012). Caspase-1 activity is required to bypass macrophage apoptosis upon Salmonella infection. Nat. Chem. Biol..

[33] Buchebner M (2010). Cholesteryl ester hydrolase activity is abolished in HSL−/− macrophages but unchanged in macrophages lacking KIAA1363. J. Lipid Res..

[34] Okazaki H (2008). Identification of neutral cholesterol ester hydrolase, a key enzyme removing cholesterol from macrophages. J. Biol. Chem..

[35] Chiang KP, Niessen S, Saghatelian A, Cravatt BF (2006). An enzyme that regulates ether lipid signaling pathways in cancer annotated by multidimensional profiling. Chem. Biol..

[36] Chang JW, Nomura DK, Cravatt BF (2011). A potent and selective inhibitor of KIAA1363/AADACL1 that impairs prostate cancer pathogenesis. Chem. Biol..

[37] Jessani N, Liu Y, Humphrey M, Cravatt BF (2002). Enzyme activity profiles of the secreted and membrane proteome that depict cancer cell invasiveness. Proc. Natl Acad. Sci. USA.

[38] Jessani N (2005). A streamlined platform for high-content functional proteomics of primary human specimens. Nat. Methods.

[39] Shaw TJ, Senterman MK, Dawson K, Crane CA, Vanderhyden BC (2004). Characterization of intraperitoneal, orthotopic, and metastatic xenograft models of human ovarian cancer. Mol. Ther..

[40] Okerberg ES (2005). High-resolution functional proteomics by active-site peptide profiling. Proc. Natl Acad. Sci. USA.

[41] Bright NA, Davis LJ, Luzio JP (2016). Endolysosomes are the principal intracellular sites of acid hydrolase activity. Curr. Biol..

[42] Simon GM, Niphakis MJ, Cravatt BF (2013). Determining target engagement in living systems. Nat. Chem. Biol..

[43] Heath JR, Ribas A, Mischel PS (2016). Single-cell analysis tools for drug discovery and development. Nat. Rev. Drug Discov..

[44] Weroha SJ (2014). Tumorgrafts as in vivo surrogates for women with ovarian cancer. Clin. Cancer Res..

[45] Weiswald LB, Bellet D, Dangles-Marie V (2015). Spherical cancer models in tumor biology. Neoplasia.

[46] Jones LH (2015). Cell permeable affinity- and activity-based probes. Future Med. Chem..

[47] Speers AE, Adam GC, Cravatt BF (2003). Activity-based protein profiling in vivo using a copper(i)-catalyzed azide-alkyne [3 + 2] cycloaddition. J. Am. Chem. Soc..

[48] Bunnage ME, Chekler EL, Jones LH (2013). Target validation using chemical probes. Nat. Chem. Biol..

[49] Nomura DK, Dix MM, Cravatt BF (2010). Activity-based protein profiling for biochemical pathway discovery in cancer. Nat. Rev. Cancer.

[50] Tully SE, Cravatt BF (2010). Activity-based probes that target functional subclasses of phospholipases in proteomes. J. Am. Chem. Soc..

[51] Moellering RE, Cravatt BF (2013). Functional lysine modification by an intrinsically reactive primary glycolytic metabolite. Science.

[52] Davidowitz RA (2014). Mesenchymal gene program-expressing ovarian cancer spheroids exhibit enhanced mesothelial clearance. J. Clin. Invest..

