# Supplementary material for: An activity-dependent proximity ligation platform for spatially resolved quantification of active enzymes in single cells
Source: Nat Commun. 2017 Nov 24;8:1775. doi: 10.1038/s41467-017-01854-0 (PMC5701173; doi:10.1038/s41467-017-01854-0)
Supplement: Supplementary file 1 — Supplementary Information [file 41467_2017_1854_MOESM1_ESM.pdf]

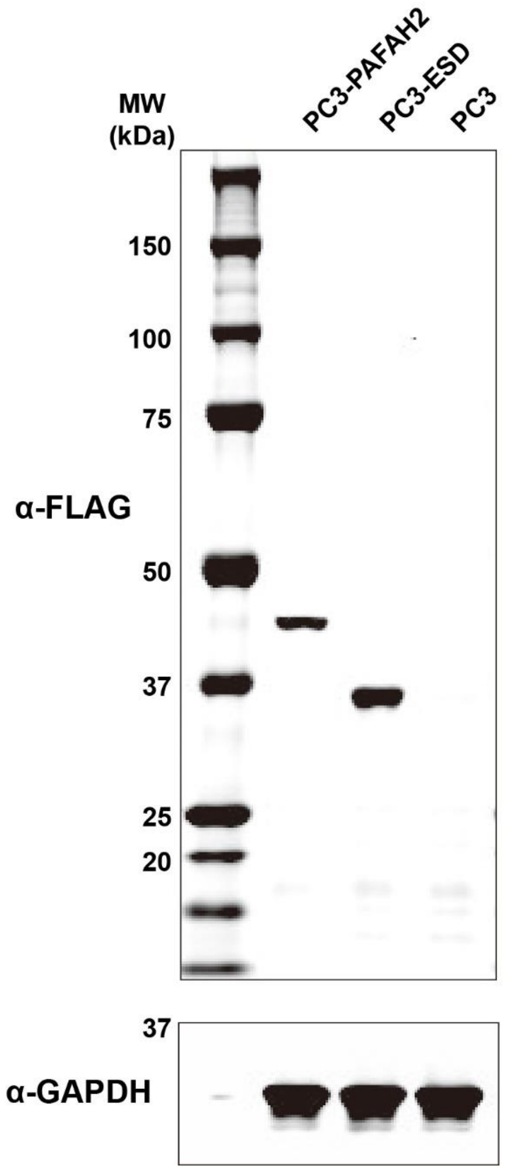

Supplementary Figure 1 | Characterization of serine hydrolase expressing stable cell lines. α-FLAG and α-GAPDH Western blots of FLAG-PAFAH2 expressing, FLAG-ESD expressing, and wild-type PC3 stable cells. Data are representative of two technical replicates in duplicate biological experiments.

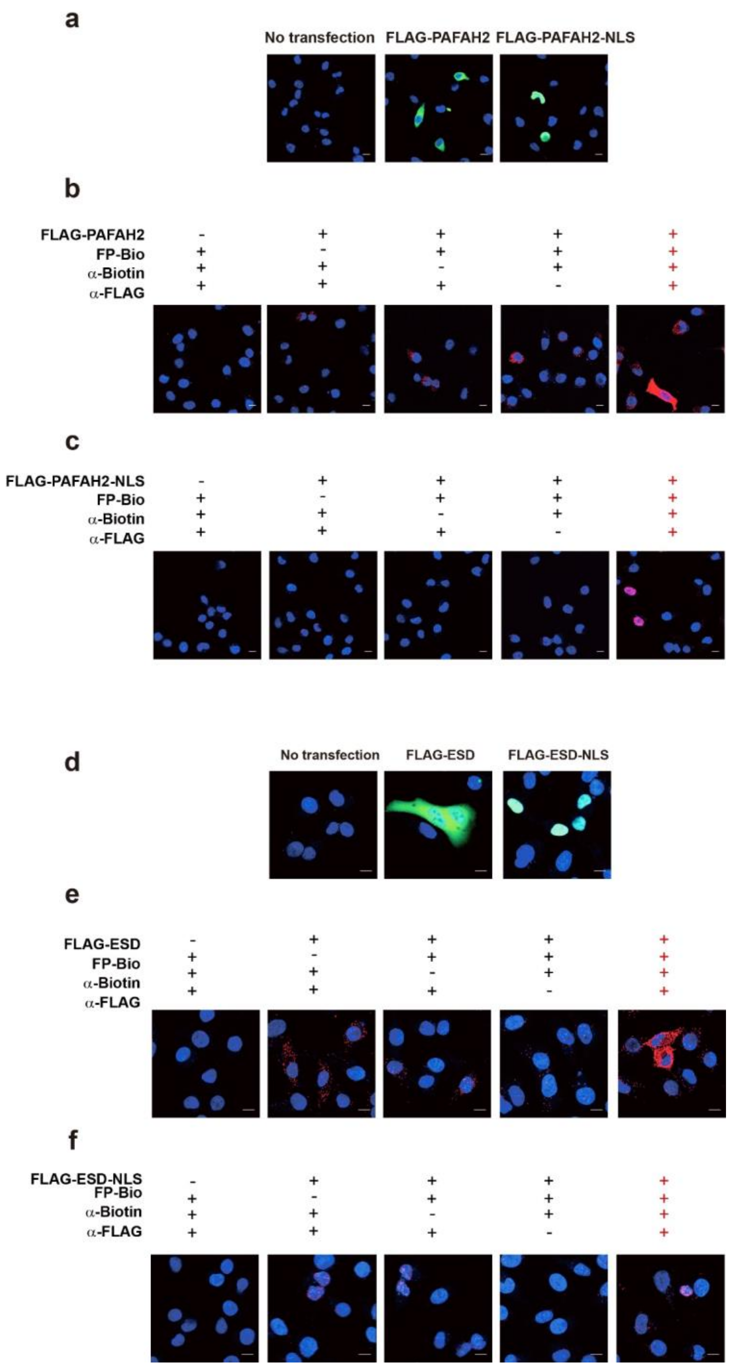

Supplementary Figure 2 | Validation of the transfection and specific detection of active serine hydrolases by ADPL imaging. (**a**) Immunofluorescence for negative transfection, FLAG-PAFAH2, NLS-PAFAH2 transfection in HeLa cells. (**b, c**) Representative ADPL detection and visualization of active FLAG-PAFAH2 (b) and NLS-PAFAH2 (c) in transfected HeLa cells in the presence or absence of indicated ADPL components. (**d**) Immunofluorescence for negative transfection, FLAG-ESD, NLS-ESD transfection in HeLa cells. (**e, f**) Representative ADPL detection and visualization of active FLAG-ESD (e) and NLS-ESD (f) in transfected HeLa cells in the presence or absence of indicated ADPL components. Channels shown are DAPI nuclear stain (blue), ADPL signal (red). Scale bars = 10 μm. Data are representative of four or more technical replicates in three or more biological replicates.

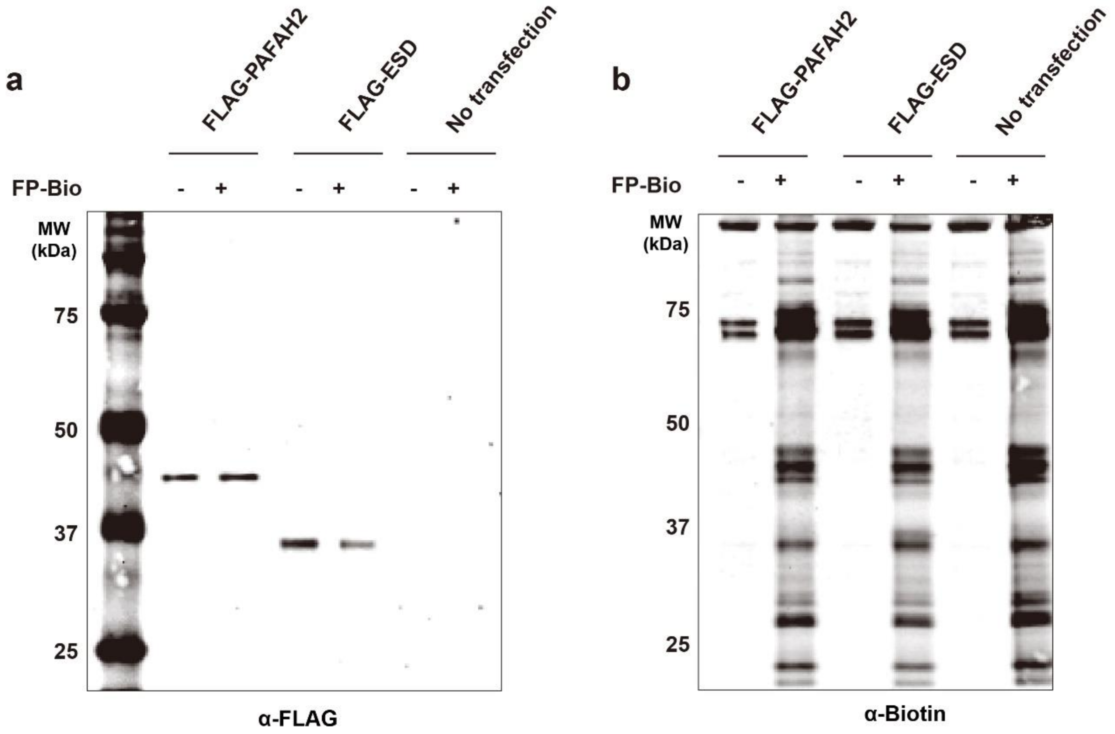

Supplementary Figure 3 | Detection of transfected active serine hydrolases in cell lysate by gel-based chemical proteomics. (**a**) α-FLAG western blot for cell lysates of FLAG-PAFAH2 transfected, FLAG-ESD transfected, and negative transfected HeLa cell lysates which were compared in pair between without probe pulsed and with probe pulsed. (**b**) α-Biotin western blot "gel-based" profiling of serine hydrolase activity in the FLAG-PAFAH2 transfected, FLAG-ESD transfected, negative transfected HeLa cell lysates which were compared in pair between without probe pulsed and with probe pulsed. Representative data are from two technical replicates of two biological experiments.

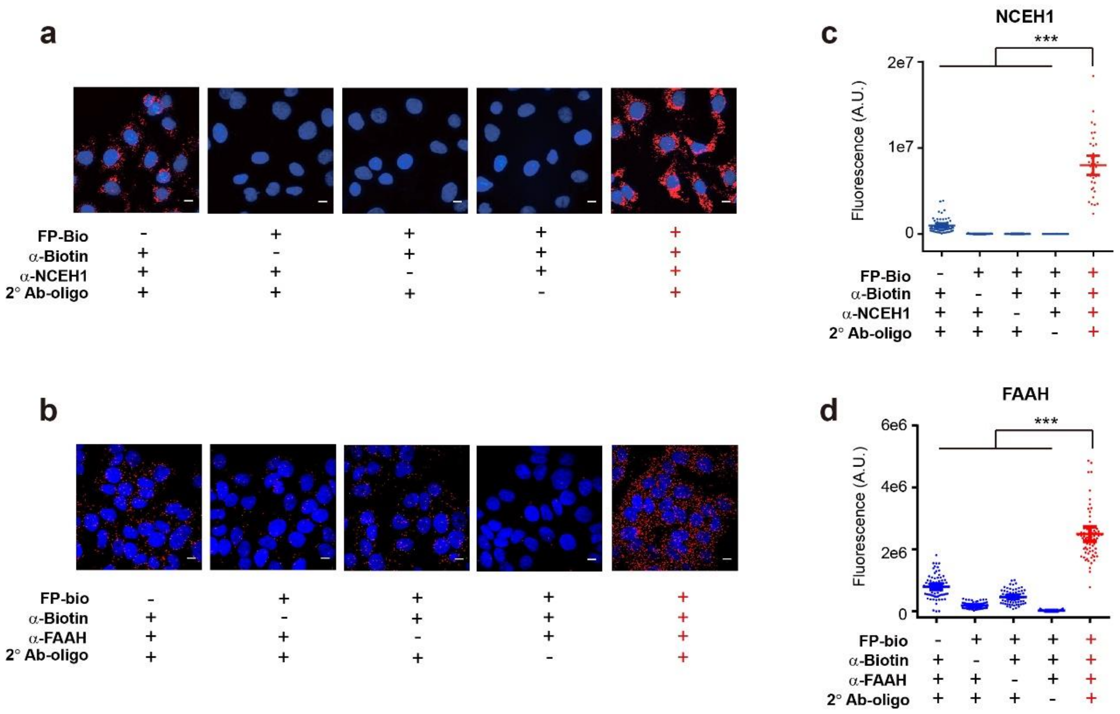

Supplementary Figure 4 | Specific detection of endogenous, active serine hydrolases NCEH1 and FAAH by ADPL imaging. (**a, b**) Representative ADPL detection and visualization of NCEH1 in SKOV3 cells (a) or FAAH in MCF7 cells (b) in the presence or absence of indicated ADPL components. Channels shown are DAPI nuclear stain (blue), ADPL signal (red). (**c, d**) Quantified single cell ADPL fluorescent signal from active NCEH1 (c) or FAAH (d) in the presence or absence of indicated ADPL components, demonstrating the probe- and POI-dependent nature of robust ADPL signal. Quantification of signal in **b**: minus FP-Bio treatment ($n = 55$), minus α-biotin ($n = 37$), minus α-NCEH1 ($n = 34$), minus 2° antibody oligo ($n = 42$), positive ADPL ($n = 38$). Quantification of signal in **d**: minus FP-Bio treatment ($n = 74$), minus α-biotin ($n = 79$), minus α-FAAH ($n = 69$), minus 2° antibody oligo ($n = 72$), positive ADPL ($n = 67$). Unpaired t-test results in **b** and **d** are between individual ADPL conditions in the absence of one component and the positive ADPL condition containing all components. ***$P < 0.001$. Each dot represents a single cell fluorescence measurement, center line and whiskers denote the mean and 95% C.I. of the population, respectively. Scale bars = 10 μm. Data are representative of four or more technical replicates in three or more biological replicates.

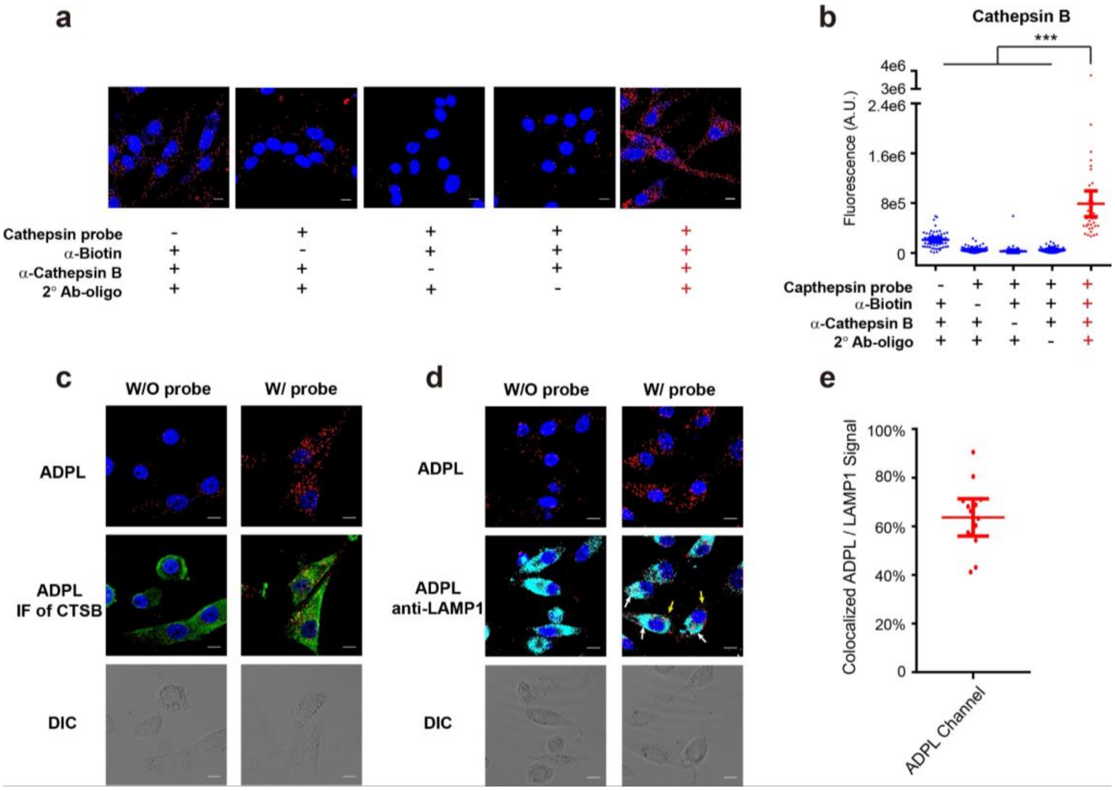

Supplementary Figure 5 | Specific detection of endogenous, active cysteine hydrolases cathepsin B by ADPL imaging and characterizing the localization of ADPL signal of cathepsin B. (a) Representative ADPL detection and visualization of cathepsin B in U87 cells in the presence or absence of indicated ADPL components. Channels shown are DAPI nuclear stain (blue), ADPL signal (red). (b) Quantified single cell ADPL fluorescent signal from active cathepsin B in the presence or absence of indicated ADPL components, demonstrating the probe- and POI-dependent nature of robust ADPL signal. Quantification of signal in b: minus FP-Bio treatment (n = 54), minus α-biotin (n = 70), minus α-cathepsin B (n = 63), minus 2° antibody oligo (n = 57), positive ADPL (n = 39). Characterizing the localization of ADPL signal of cathepsin B in U87 cells. (**c**) Co-localization analysis of ADPL and immunofluorescence detection of cathepsin B without (w/o) and with (w/) probe. (**d**) Co-localization analysis of ADPL and lysosome marker anti-LAMP1 Alexa Fluor® 647 without (w/o) and with (w/) probe. White arrows indicate the co-localized ADPL signal; yellow arrows indicate the signal outside LAMP1-stained lysosomes. (**e**) Quantification of the percentage of ADPL signal in lysosome on a per-cell basis. Unpaired t-test results in **b** are between individual ADPL conditions in the absence of one component and the positive ADPL condition containing all components. ***P < 0.001. Each dot represents a single cell fluorescence measurement, center line and whiskers denote the mean and 95% C.I. of the population, respectively. Scale bars = 10 μm. Data are representative of four or more technical replicates in three or more biological replicates.

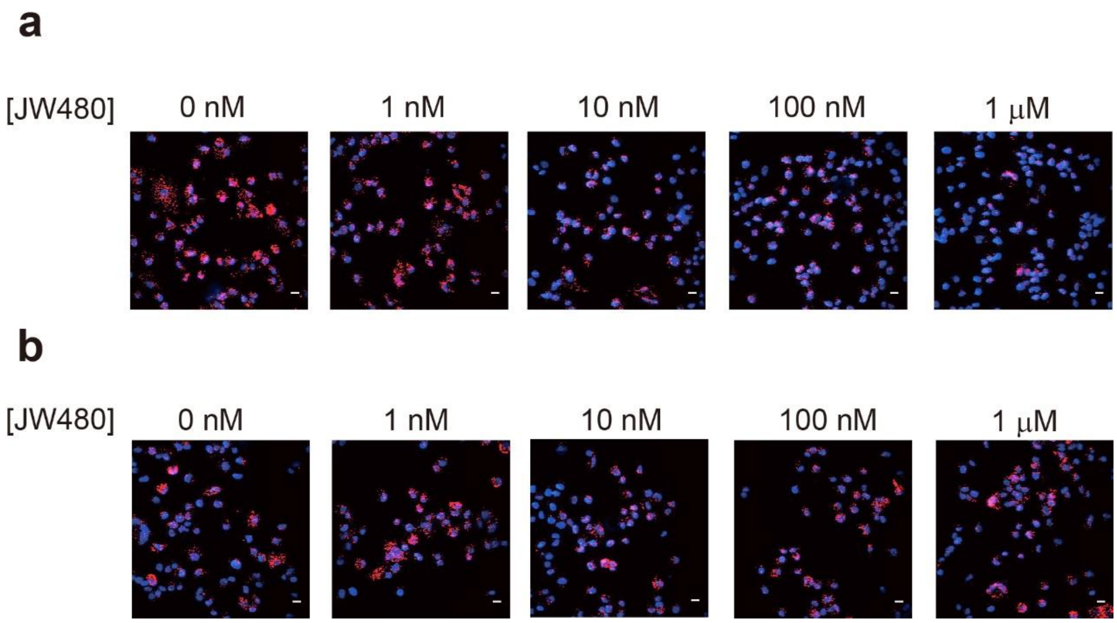

Supplementary Figure 6 | ADPL detects active enzymes and the specific action of small molecule inhibitors. (**a, b**) Representative ADPL images of NCEH1 (a) and PAFAH2 (b) activity in PAFAH2-expressing PC3 stable cells with FP-Bio labeling following pre-treatment of live cells with the specific NCEH1 inhibitor JW480 at the indicated concentration for 4 hours. Scale bars = 20 μm in all images. Blue channel: DAPI nuclear; red channel: ADPL signal. Images are representative of four technical replicates in duplicate biological experiments.

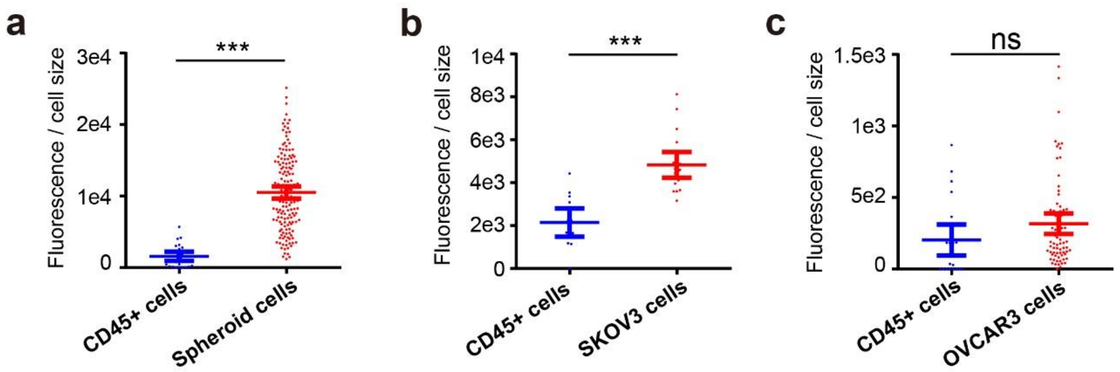

Supplementary Figure 7 | Area-normalized ADPL quantification of endogenous enzyme activity in cellular co-culture and primary patient samples. (**a-c**) Cell area-normalized quantification of NCEH1 activity in primary ovarian cancer spheroids (a), SKOV3 ovarian cancer cells (b) and OVCAR3 ovarian cancer cells (c) and primary immune cells. Quantification in **a**: immune cells ($n = 25$), cancer cells ($n = 155$). Quantification in **b**: immune cells ($n = 14$), SKOV3 cancer cells ($n = 20$). Quantification in **c**: immune cells ($n = 23$), OVCAR3 cancer cells ($n = 74$). Each point represents a single cell fluorescence measurement, center-line and whiskers denote the mean and 95% C.I. of the population; unpaired student t-test was used for statistical significance. ***, $P < 0.001$; ns, not significant. Data are from four or more technical replicates from independent duplicate biological experiments.

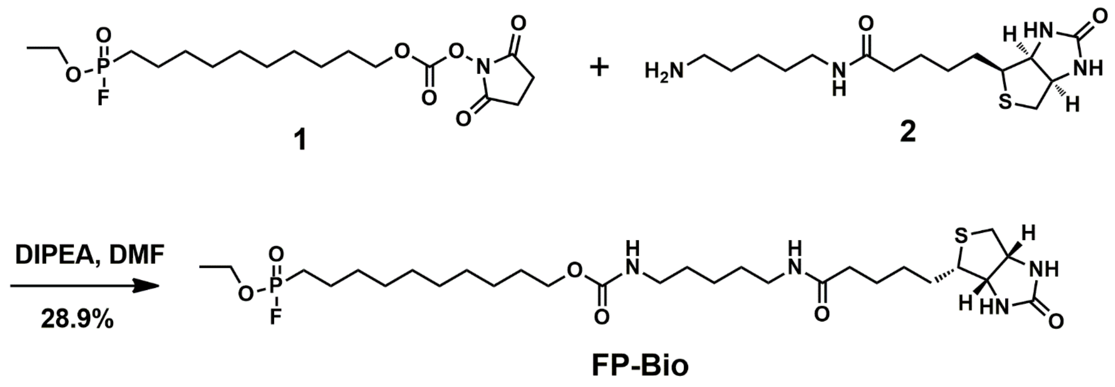

Supplementary Figure 8 | Fluorophosphonate-biotin (FP-bio) probe synthesis.
